# Variation in cultural attitude, knowledge and individual motivational factors impact engagement and tool use in a field experiment in wild chimpanzees

**DOI:** 10.1098/rsos.261322

**Published:** 2026-10-21

**Authors:** Kelly R. Mannion, Michael Ndora, Stephen Kugonza, Thibaud Gruber

**Affiliations:** ^1^ Swiss Centre for Affective Sciences, University of Geneva, Geneva, GE, Switzerland; ^2^ Faculty of Psychology and Educational Sciences, University of Geneva, Geneva, GE, Switzerland; ^3^ Bugoma Primate Conservation Project, Uganda

**Keywords:** tool use, culture, experiments, *Pan troglodytes*, cultural attitude, interindividual variation

## Abstract

Cultural traditions shape how animals approach ecological problems. Over the last two decades, Ugandan chimpanzees (*Pan troglodytes schweinfurthii*) have engaged with the honey-trap experiment, an apparatus mimicking a beehive, while relying on their cultural knowledge. Here, we compared the behaviour of three Ugandan chimpanzee communities in the experiment, adding a previously untested community, the Mwera South chimpanzees of Bugoma Forest. First, we tested whether communities varied in their engagement with the experiment and investigated whether differences lay in individual or community-based characteristics. Second, we analysed individual variation within the same culture, particularly focusing on the new data coming from Bugoma Forest. We found that only the Kanyawara chimpanzees of Kibale Forest displayed a dedicated instrumental method, stick use, realized in their cultural knowledge and expressed by most members of the community, congruent with the largest engagement with the resource and suggestive of a general positive cultural attitude towards honey. By contrast, the two other communities appeared to lack a cultural attitude towards honey, with no attached tool technique overall. Nevertheless, there were also large inter-individual differences. Notably, Bugoma chimpanzees displayed undescribed behavioural flexibility, using a range of tool behaviours, including both stick and leaf tool use, a pattern never documented before. Cultural attitudes toward resources thus constitute an additional layer to culture in addition to instrumental knowledge and coexist with individual motivational traits in influencing the realization of cultural behaviour.

## 1. Introduction

Decades of research in established long-term field sites yield a plethora of evidence in support of a growing number of animal species having culture. In particular, over the last two decades, evidence for social learning has been found across many taxa, whether in avian, mammalian or even insect species, and in a range of behaviours, such as foraging or vocalizations [1–5]. Specifically, these cultural species have displayed the potential and ability for social learning that enables the transmission of behavioural traditions that are passed on through generations [6–8]. Correspondingly, research has also looked into whether and how, as in humans, an individual's culture influences their behaviour in nature or in the context of field experiments [9].

This cognitive approach to culture has been complemented in recent years by wider approaches to what we can consider cultural in animals. For example, Schuppli & van Schaik [10] argue that by focusing only on remarkable behaviour, such as tool use, we ‘underestimate culturally transmitted basic subsistence skills’, such as revealed in diet [11]. Others have argued for differences in what can be characterized as *social attitudes* to count as cultural variants (e.g. [12,13]). The two approaches are not necessarily incompatible. For example, a particular attitude towards a specific food resource may either favour or disfavour its consumption within the population. Many examples are found in humans, sometimes based on religious norms, but also generally building ground for culinary cultures.

The latter points illustrate that culture is multi-faceted, including the learning of *values* [14,15], the resulting modification of one's attitude towards a particular stimulus (from neutral to the point of ignoring it to being interested and attributing valence to that stimulus), as well as the potential learning of an instrumental action towards that stimulus, such as learning a tool use technique to access it. The modification of one's attitude towards a given food substrate commonly eaten in a community influenced by social learning will be here referred to as *cultural attitude*, compared with the social learning of instrumental actions, such as tool use, which will be referred to as *cultural knowledge*.

Crucially, the two notions intersect. How individuals within a community implement a cultural attitude can vary, particularly if a given instrumental action is connected to it. For example, if a particular resource is highly valued in the community and there is a dedicated technique to access it, one can predict that this technique will prevail in the community. The reliance on this technique to acquire the given resource may, in turn, be gradually improved to maximize output [16]. On the contrary, if there is only a weak interest in a given object or resource, there may neither be a set technique dedicated to obtaining the latter nor selection for more efficient techniques. As such, we can predict that communities where there is only a weak interest for a given resource will not have a set attached behaviour towards the food reward. This, however, does not exclude individual preferences, and as such does not preclude some individuals from the community from having a certain knack for the object of interest and making use of their existing knowledge, cultural or not, to obtain that resource.

The Eastern African chimpanzee (*Pan troglodytes schweinfurthii*) Ugandan chimpanzee populations appear particularly suited for testing questions related to cultural attitudes and individual differences regarding food items. Cultural repertoires, including tool use behaviour, have been described for several Ugandan chimpanzee communities owing to long-term field sites with research and conservation projects. The innovation and subsequent transmission of moss-sponging to acquire water has been well documented in the Sonso community of Budongo Forest [2,17]. However, unlike most other chimpanzee communities, the Budongo chimpanzees have not been recorded using stick tools (for a review see [18]). Stick use is, however, described throughout Uganda, including in Kibale Forest [19], Kalinzu Forest [20] and in neighbouring forest patches to Budongo, such as Kasongoire or Bulindi [21]. The distinction in stick tool use between Kibale and Bulindi chimpanzees is also of interest with respect to cultural knowledge: Kibale chimpanzees use stick tools to probe, whereas Bulindi chimpanzees use stick tools to dig in the ground and unearth beehives [22].

To test hypotheses in the field rather than a laboratory or captive context, researchers have implemented field experiments on wild populations, which deliver more ecological validity compared with semi-wild or captive contexts [23–25]. Field experiments testing foraging behaviour can be used to examine cultural tendencies, such as tool use in foraging scenarios, for example, by presenting a substrate and the required tool material [26,27]. For example, Gruber *et al.* [28,29] developed the honey-trap experiment, which simulates a natural context, such as a beehive, where the honey is only accessible through a small hole, thus presenting the opportunity for the chimpanzees to use a tool to extract the honey. Chimpanzees from the Budongo and Kibale forests displayed community-specific responses to extract honey from the apparatuses, relying on their already established foraging tool use behaviour. Crucially, they did not develop behaviour outside of their cultural knowledge. More recently, Koops *et al.* [24] replicated these results in another subspecies, Western African chimpanzees (*Pan troglodytes verus*), and with a different tool behaviour: nut-cracking using stones. Combined, the results of these studies suggest that foraging field experiments have little impact on triggering or modifying wild chimpanzee behaviour, beyond multiplying opportunities for chimpanzees to express somewhat rare behaviour without modifying their cultures, which would constitute an ethical issue [30]. As such, they can rather be seen as a revelator of the current cultural behaviour of chimpanzees. In addition, by solely providing opportunities for engagement, field experiments may also act as a proxy for general interest in the resource. For example, the Kanyawara chimpanzees of Kibale Forest appeared to display general differences in their interest in honey compared with the Sonso chimpanzees of Budongo Forest [18].

While variation in tool use across Ugandan communities is well documented and thus makes the study of chimpanzee feeding cultures there well grounded, some aspects are still unknown. The Bugoma Forest lies in the middle between Kibale and Budongo forests, with the resident chimpanzee communities exhibiting marked cultural differences in terms of their nest building behaviour [31,32]. Yet, their foraging cultural toolkit remains elusive, with possible observations of stick use being suggestive of a stick tool culture (supplementary material, figure S2). These early observations thus suggested that the cultural knowledge of the Bugoma chimpanzees would be closer to that of Kibale chimpanzees. At the same time, the documented behaviour of the Mwera South community of Bugoma Forest during habituation suggested that their attitude towards honey appeared closer to that of the Sonso chimpanzees of Budongo Forest compared with that of the Kanyawara chimpanzees of Kibale Forest (Gruber's personal observations). Therefore, the addition of this third chimpanzee community allowed triangulating the aspects of interest, attitude and instrumental means towards a resource as described above, with the Mwera South and Kanyawara chimpanzees appearing to share the same instrumental means to reach honey (stick tools) and ecological conditions [33], but with the Mwera South and Sonso chimpanzees appearing to share the same attitude towards honey (less general interest compared with Kanyawara chimpanzees).

Here, we presented the resident Mwera South chimpanzees of Bugoma Forest with variations of the honey-trap experiment to allow them to express their foraging strategies in a controlled environment. We replicated two previous studies with the Sonsoo and Kanyawara chimpanzees to offer the Mwera South chimpanzees the same ecological affordances as previously offered to the other two communities. In particular, we both presented the standard honey-trap experiment (a hole carved into a large log filled with honey and covered by honeycombs [28]) and the same setting complemented with offering a leafy-stick tool [29]. We first compared the results of the experiment across the three communities and showed that the Kanyawara chimpanzees generally displayed more interest towards honey compared with the Sonso and Mwera South chimpanzees. This suggested that there are community-level differences in terms of attitude towards a given resource and was combined with a unique reliance on a specific tool-using technique. Nevertheless, in all communities we found individuals who appeared to be particularly keen to access the resource. These chimpanzees were also the ones who used the largest diversity of means to access honey, including tool techniques. In particular, we observed undescribed flexibility in tool use in individuals from the Mwera South community, including one male chimpanzee who displayed both stick manufacturing and leaf-sponging to solve the problem. We discuss the interaction between community-specific factors and individual factors, such as motivation in chimpanzees expressing their tool-using behaviour.

## 2. Methods

### 2.1. Study site and subjects

Bugoma Central Forest Reserve, Uganda (01°15′ N 30°58′ E) is a semi-deciduous tropical rain forest located between the Central Forest Reserve of Budongo (425 km^2^) and Kibale National Park (776 km^2^). The Bugoma Primate Conservation Project (BPCP, www.bugomaprimates.com) has been studying the Mwera South community since 2016, with habituation and identification of individuals in the community still ongoing. Currently, the community is estimated to have about 70 individuals, similar to other East African chimpanzee community sizes. Research efforts with the Mwera South chimpanzees have already established the use of leaf-sponging on a regular basis to obtain water and leaf napkins/wipes (see supplementary material, videos), as well as the existence of specific cultural behaviour, such as ground nesting [31]. Because habituation was ongoing at the time of the study, it was unknown prior to the experiments what other tool use behaviour the Bugoma chimpanzees had in their repertoire.

The Sonso community (01°43′ N, 31°32′ E) inhabits the Budongo Forest Reserve, a 482 km² area of continuous medium-altitude semi-deciduous forest at approximately 1050 m elevation. Habituation of the Sonso community began in 1990 and was considered complete by the time of the experiments. The Kanyawara community (00°33′ N, 30°21′ E), located approximately 180 km from Sonso in Kibale National Park, has been continuously studied since 1987 and was fully habituated by the time of the experiments. Both communities practice leaf-sponging. Notably, Sonso chimpanzees do not display any other foraging-related tool use [2]. Kanyawara chimpanzees also exhibit fluid-dip behaviour habitually. Data in the honey-trap experiment for these communities came from studies by Gruber *et al.* [28,29] in the obligatory setting. In that setting, a hole of 5 × 4 cm was drilled, with honey filled up to 10 cm from the surface, covered by honeycombs as visual attractors.

### 2.2. Experimental set-up

The set-up is only described for Bugoma in the current study. For data in Budongo and Kibale, please refer to Gruber *et al.* [28,29] and Gruber [18].

Logs from fallen trees were collected from the forest and used to create experimental apparatuses. We cut a rectangular-shaped hole (4 × 5 × 16 cm) into them using a hammer and chisel. After creating the hole, experiment logs were always disinfected and left to rest in a nearby clearing prior to being set up in the forest. Any personnel who handled the experiment apparatus or equipment for set-up were required to wear face masks and gloves to avoid contamination (see [30] for ethical aspects of field experiments, including risks for disease transmission).

The prepared logs served as the basis of the honey-trap experiment. Honey was poured into the hole in the log once set up in the forest until there was a 10 cm gap from the top of the honey in the hole to the surface of the log, replicating Gruber *et al.* [28] setting (obligatory condition). Afterwards, pieces of honeycomb were placed on top of the hole (figure 1) and held in place by beeswax to help provide a visual cue to the chimpanzees as well as deter bees and other insects from accessing the honey. There were no additional means used to attract individuals or encourage them to engage with the experiment. Honey, honeycombs and beeswax for the experiments were harvested from hives maintained by local beekeepers in the villages surrounding Bugoma Forest Reserve. The bees were of the genus *Apis* and could move freely within and around the forest area.

**Figure 1.**
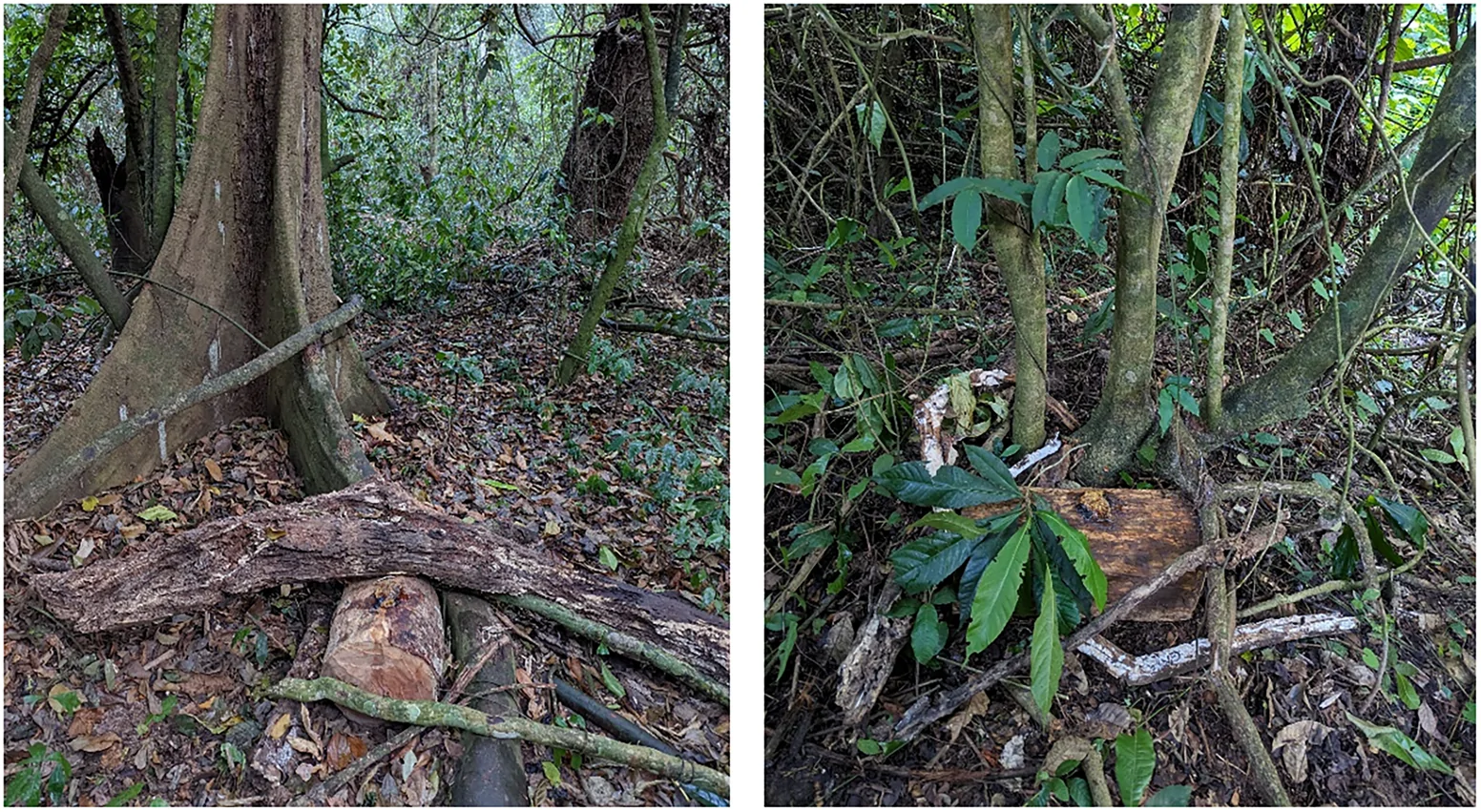
(Left) honey-trap experiment 1. Honeycomb held in place over the hole with beeswax. The experiment is fixed to the tree buttress. (Right) honey-trap experiment 2 with a leafy stick tool provided.

As habituation was ongoing at the time of the experiments, we did not follow chimpanzees carrying a log as in Gruber *et al.* [29] but installed it in a location we predicted chimpanzees would pass by, based on their foraging activity at the time of the experiment setting. Trails and resting areas near feeding trees were used depending on the frequency with which the chimpanzees were using these areas during their normal day-to-day routine. Experiment areas preserved the normal forest underbrush and leaf litter present, ensuring the most naturally simulated context and providing potential raw materials for the chimpanzees to use.

Multiple motion-sensing camera traps (Bushnell CoreDS) were set up around the experiment, ensuring the visibility of the experiment log and unobstructed angles of individuals that engaged with it. Each experiment was set up prior to the chimpanzees arriving in the area to ensure that researchers would not be associated with the experiment. After setting up the experiment, researchers left the area and avoided any interaction with the chimpanzees. The experiment was left in place for a maximum of 10 days, with the researchers refilling the honey every morning.

Experiment 1 consisted of the honey-trap experiment in the obligatory condition as in Gruber *et al.* [28,29]. Experiment 2 consisted of the honey-trap in the obligatory condition experiment with the addition of a leafy stick laid on the top of the experiment log between the hole and the log edge to serve as a potential tool with multiple functions [29]. The side of the experiment log on which the tool was placed changed randomly with the experiment trials. The potential leafy stick tool was a 40 cm branch/sapling of *Alstonia* sp., which is commonly found in Bugoma Forest. The lower 20 cm portion of the tool had the leaves removed so that the tool could be used in different ways: to dip into the apparatus using just the stick end without leaves, to leaf-sponge by removing leaves to chew into a clump to dip into the hole and extract the honey like using a sponge or as a combined leaf-dipping tool by inserting the leafy end of the stick and sucking honey off the attached leaves that were dipped into the hole. The latter behaviour was never displayed by either the Kanyawara or Sonso chimpanzees (see [18] for a review). This experimental set-up tests whether chimpanzees can identify a potentially useful tool to extract the honey.

Between May 2021 and November 2022, we focused on experiment 1, while in 2024, we focused on experiment 2. The research was reviewed and received approval from the Uganda Wildlife Authority (permit no. COD-96-05 to K.R.M.) and the Uganda National Council of Science and Technology (permit no. NS155ES to K.R.M.).

### 2.3. Video data in Bugoma Forest

Data were extracted from the videos using BORIS software [34]. Identities of chimpanzees and classification of behaviours were coded by creating a customized ethogram specific for behaviour concerning wild chimpanzees in a foraging context (table 1). Data recorded from the videos were exported from BORIS as a .csv file and imported into RStudio for further processing and analyses.

**Table 1.** Ethogram to classify the behaviour exhibited by the Mwera South chimpanzees exhibited during honey-extraction and the more general groups they were classified in.

			leaf sponge	mashing leaves in mouth then using that to dip into the experiment hole and bring out honey
		tool use	honeycomb sponge	using the honeycomb that was covering the experiment hole to dip into it and extract hole
	honey extraction		leaf dip	a stick with leaves being used to insert in experiment hole and extract honey
			stick tool	a stick with no leaves being used to extract honey
direct engagement			fingers in hole	using fingers to reach into experiment hole to extract honey
			feeding honeycomb	feeding on honeycomb that was placed over experiment hole
			honeycomb interaction	picking at and touching honeycomb while it is covering the experiment hole
			active engagement	interacting physically with the experiment log but not with the hole for example: biting, licking and pulling
indirect engagement			observing experiment	looking at experiment apparatus
			peering	watching another individual in close proximity to see what they are doing at the experiment
			smelling experiment	smelling the experiment apparatus or hole
			touching experiment	in contact with the experiment but not looking at it or doing anything with it, for example sitting on it

### 2.4. Data analysis

Data for Bugoma were directly coded from the video data by K.R.M. Data for the honey-trap experiment from the Sonso community in Budongo and Kanyawara community in Kibale were extracted from Gruber *et al.* [29, table 1], ‘Baseline’ experimental condition with non-zero total interaction time, and compared with the data acquired from the Mwera South community in Bugoma in the present study. All data duration in Mwera South were coded to the ms on BORIS and analysed as such in statistical analyses, but rounded to seconds in tables and figures for comparison purposes. Overall, we present data for N_Sonso_ = 34, N_Kanyawara_ = 15 and N_Mwera–South_ = 16 chimpanzees. An outlier was considered an individual whose engagement time was less than or more than 3 s.d. from the overall mean across all communities (pooled threshold). This identified two individuals: QT from Kanyawara (z-score = 5.9) and HON from Mwera South (z-score = 3.6). In addition, while not qualifying as outliers, a few individuals in Sonso (*N* = 5) and Mwera South (*N* = 6) engaged for less than 20 s with the experiment. The 20 s mark was originally taken as a threshold in Gruber *et al.* [29] for displaying tool use, as this was the median time before which Kanyawara chimpanzees displayed tool use in the original study [28,29].

### 2.5. Statistical analyses

We compared total community engagement by conducting quantile regressions to the 50% quantile (i.e the median) because this method is robust to outliers and adapted to our dataset, which consisted of pooled duration across individuals and communities. To compare with previously published comparisons between Sonso and Kanyawara in Gruber *et al.* [29], which suggested no significant difference between the two communities in engagement time, we first fitted a quantile regression, including the effect of community only (marginal community effect). Following this, we fitted a quantile regression additionally adjusted for the following covariates: tool use (yes/no), sex (F/M), age class (adult, sub-adult and juvenile) and number of encounters (adjusted community effect). Indeed, while there are documented differences in tool use among chimpanzees, for example, across sex [35], we did not have specific predictions besides potential community differences at the onset of this study. Finally, we fitted a quantile regression to the 90% quantile, which allowed us to model community differences for outlying engagement values, and which was a departure from the original comparison, including only Sonso and Kanyawara.

All analyses and raw data can be found at the following repository link: https://github.com/ChimpanzeeResearch/bugoma_tool_use/tree/main.

## 3. Results

### 3.1. Comparison of engagement in the honey-trap experiment across field sites

Table 2 summarizes the results for engagement time in the honey-trap experiment by the three communities as well as information regarding the individuals themselves. There was no effect of community at the median (*F*(2,62) = 0.66, *p* = 0.52) or at the 90% quantile (*F*(2,62) = 0.76, *p* = 0.47) for the marginal quantile regressions. For the adjusted quantile regressions, we found an effect of community at the 90% quantile (*F*(2,57) = 7.50, *p* = 0.0012): chimpanzees in Kanyawara spent more time engaging with the honey-trap compared with both Mwera South (*t*(1) = 3.562, *p* = 0.008) and Sonso (*t*(1) = 3.375, *p* = 0.0013), but there was no difference between Mwera South and Sonso (*t*(1) = –1.336, *p* = 0,1869). In addition, some of the covariate effects in the adjusted models were also significant. At the median, we found an effect of tool use (*F(*1,7) = 10.71, *p* = 0.0018), with individuals having used a tool spending more time engaging with the experiment than those who had not. At the 90% quantile, adults engaged more with the experiment than other age classes (*F*(1,57) = 3.90, *p* = 0.026), and, unsurprisingly, individuals with more encounters engaged significatively longer with the honey-trap experiment (*F*(1,57) = 30.63, *p* < 0.0001). These results are summarized in table 2.

**Table 2.** Summary statistics of comparison between Kanyawara, Mwera South and Sonso during the baseline condition, experiment 1.

quantile marginal models	community effect	*F*	DF1	DF2	*p*
0.5	covariate: none	0.66	2.00	62.00	0.5214
0.9	covariate: none	1.07	2.00	57.00	0.3493
***adjusted models***					
0.5	community	1.07	2.00	57.00	0.3493
0.5	sex	0.12	1.00	57.00	0.7336
0.5	age	0.02	2.00	57.00	0.9808
0.5	encounters	0.86	1.00	57.00	0.3581
0.5	tool (binary)	10.71	1.00	57.00	**0.0018**
0.9	community	7.50	2.00	57.00	**0.0012**
0.9	sex	0.00	1.00	57.00	0.9683
0.9	age	3.90	2.00	57.00	**0.0258**
0.9	encounters	30.63	1.00	57.00	**0.0000**
0.9	tool (binary)	0.70	1.00	57.00	0.4069

In addition, there were noticeable qualitative differences between the communities: 13 of 15 Kanyawara chimpanzees used a stick in the experiments (86.7%), with none using a different tool, compared with five Sonso chimpanzees of 34 (14.7%) manufacturing a leaf-sponge (none used a stick). Finally, three Mwera South chimpanzees of 16 (18.8%) used a variety of tool strategies (including stick use, brush use and leaf-sponges, see below).

### 3.2. Inter-individual variation

As shown in table 3 and figure 2, there was considerable within-community variation in engagement times, particularly in Kanyawara and Mwera South, while Sonso showed a more compressed distribution with 75% of values falling below 105 s. Kanyawara chimpanzees, while generally relying on the same tool-using technique, showed the largest variation in engagement time. The Mwera South chimpanzees are described below in more detail.

**Table 3.** Summary of individual engagement from chimpanzees during experiment 1 of the honey-trap experiment across three Ugandan communities. *: individuals that represent statistical outliers. Engagement time shown has been rounded to the nearest integer.

community	id	sex	age class	direct engagement (s)	tool	number of encounters
Kanyawara	AL	F	adult	138	stick	3
Kanyawara	AT	M	juvenile	259	stick	2
Kanyawara	AZ	M	juvenile	460	stick	1
Kanyawara	BO	M	juvenile	97	stick	1
Kanyawara	ES	M	sub-adult	286	stick	1
Kanyawara	EU	F	juvenile	846	stick	2
Kanyawara	KK	M	adult	72		1
Kanyawara	LR	F	adult	502	stick	1
Kanyawara	NP	F	sub-adult	2899	stick	4
Kanyawara	OG	M	juvenile	935	stick	2
Kanyawara	OT	F	sub-adult	352	stick	4
Kanyawara	PG	M	adult	49		3
Kanyawara	QT*	F	adult	7280*	stick	11
Kanyawara	TJ	M	adult	1540	stick	1
Kanyawara	TT	M	juvenile	44	stick	1
Sonso	AN	F	sub-adult	30		1
Sonso	FD	M	sub-adult	5		1
Sonso	FK	M	juvenile	10		1
Sonso	HL	F	sub-adult	98		1
Sonso	HT	F	adult	102	leaves	1
Sonso	HW	M	adult	381	leaves	5
Sonso	JT	F	juvenile	121		1
Sonso	KA	F	juvenile	20		1
Sonso	KE	F	sub-adult	10		1
Sonso	KL	F	adult	5		1
Sonso	KR	F	juvenile	30		1
Sonso	KS	M	juvenile	235	leaves	1
Sonso	KW	F	adult	78		3
Sonso	KY	M	adult	335		1
Sonso	KZ	M	adult	225		3
Sonso	ML	F	adult	45		1
Sonso	MN	F	juvenile	45		1
Sonso	MS	M	adult	335		1
Sonso	NB	F	adult	1547		4
Sonso	NR	F	sub-adult	391		5
Sonso	NT	F	juvenile	1738	leaves	5
Sonso	RS	F	sub-adult	1082	leaves	3
Sonso	SB	F	adult	360		3
Sonso	SE	F	adult	45		1
Sonso	SM	M	adult	45		1
Sonso	SQ	M	adult	34		3
Sonso	TK	M	adult	267		2
Sonso	VR	F	sub-adult	115		1
Sonso	WL	F	adult	102		1
Sonso	ZD	M	juvenile	33		1
Sonso	ZF	M	adult	18		1
Sonso	ZG	M	sub-adult	119		3
Sonso	ZL	M	adult	287		5
Sonso	ZM	F	adult	25		2
Mwera South	HON*	F	adult	4640*	leaves	13
Mwera South	ENG	M	adult	1679	stick & leaves	5
Mwera South	BRO	M	adult	1151		3
Mwera South	KBY	M	adult	1048		3
Mwera South	MAM	F	adult	876	leaves	9
Mwera South	ARP	M	adult	462		4
Mwera South	JUV	M	juvenile	60		10
Mwera South	AFO	F	adult	34		4
Mwera South	SAM	M	sub-adult	33		3
Mwera South	CAP	M	adult	29		5
Mwera South	AM2	M	adult	18		5
Mwera South	KAT	M	juvenile	18		3
Mwera South	MKU	M	adult	7		3
Mwera South	GER	M	adult	7		2
Mwera South	AF2	F	adult	4		6
Mwera South	JUVF	F	juvenile	2		2

**Figure 2.**
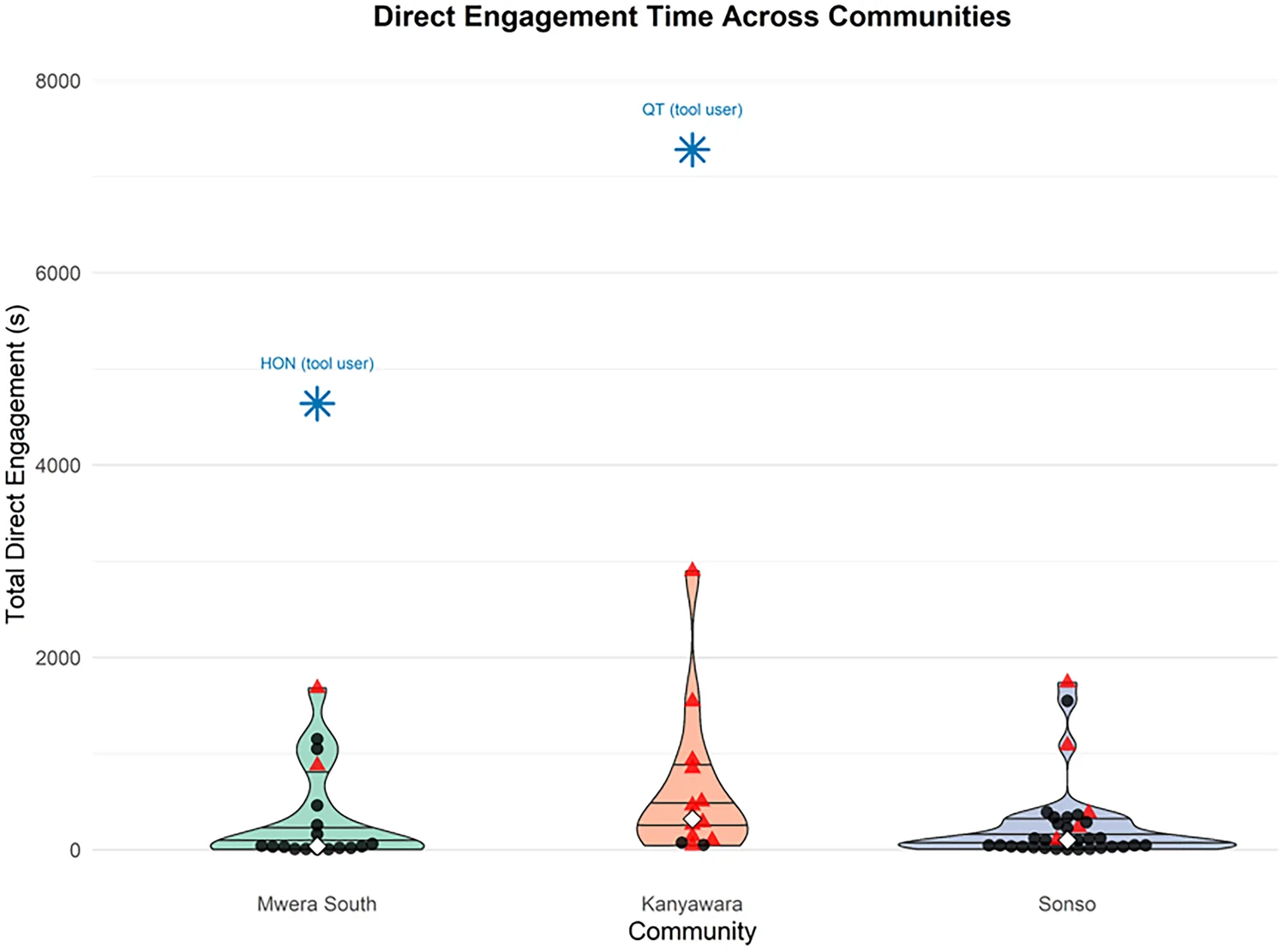
Distribution of total direct engagement time (in seconds) across three chimpanzee communities. Violin plots show the probability density of the data at different values, with individual data points overlaid (black dots for non-tool users and red triangles for tool users, stars for outliers, with mention of their tool-using status). The width of each violin represents the kernel density estimation of the underlying distribution. White diamonds indicate the median of each group. Kanyawara shows the highest median engagement time (352 s; N_Kanyawara = 15), followed by Sonso (100 s; N_Sonso = 34) and Mwera South (33.5 s; N_Mwera = 16), though with considerable overlap in their distributions. Data for Kanyawara and Sonso are taken from Gruber *et al.* []. Both statistical outliers in Mwera South and Kanyawara are tool users.

We conducted a total of nine baseline experiments in Bugoma Forest with no tool provided, i.e. experiment 1, which led to 2:55:22 (hours:minutes:seconds) of interaction by more than 16 individuals across 30 days (from a total of 46 days that had some presence of chimpanzees at the experiment). Most individuals interacted by trying to use their fingers to reach the honey at the bottom of the hole or by biting or moving the experiment apparatus with brute force to get the honey. Of those that interacted with the experiment, only three individuals used a tool to successfully extract the honey, resulting in 16.16% of their total engagement time (see figure 3 for exact breakdown). When specifically looking at honey extraction behaviours, chimpanzees relied on tool use 19.41% of the time and tried using their fingers the other 80.59% of the time (see figure 3 for breakdown and table 4 for behaviours). Interestingly, we recorded four separate types of tool-using behaviours by three individuals: stick tool, leafy-stick dip, leaf sponge and honeycomb sponge (table 4).

**Figure 3.**
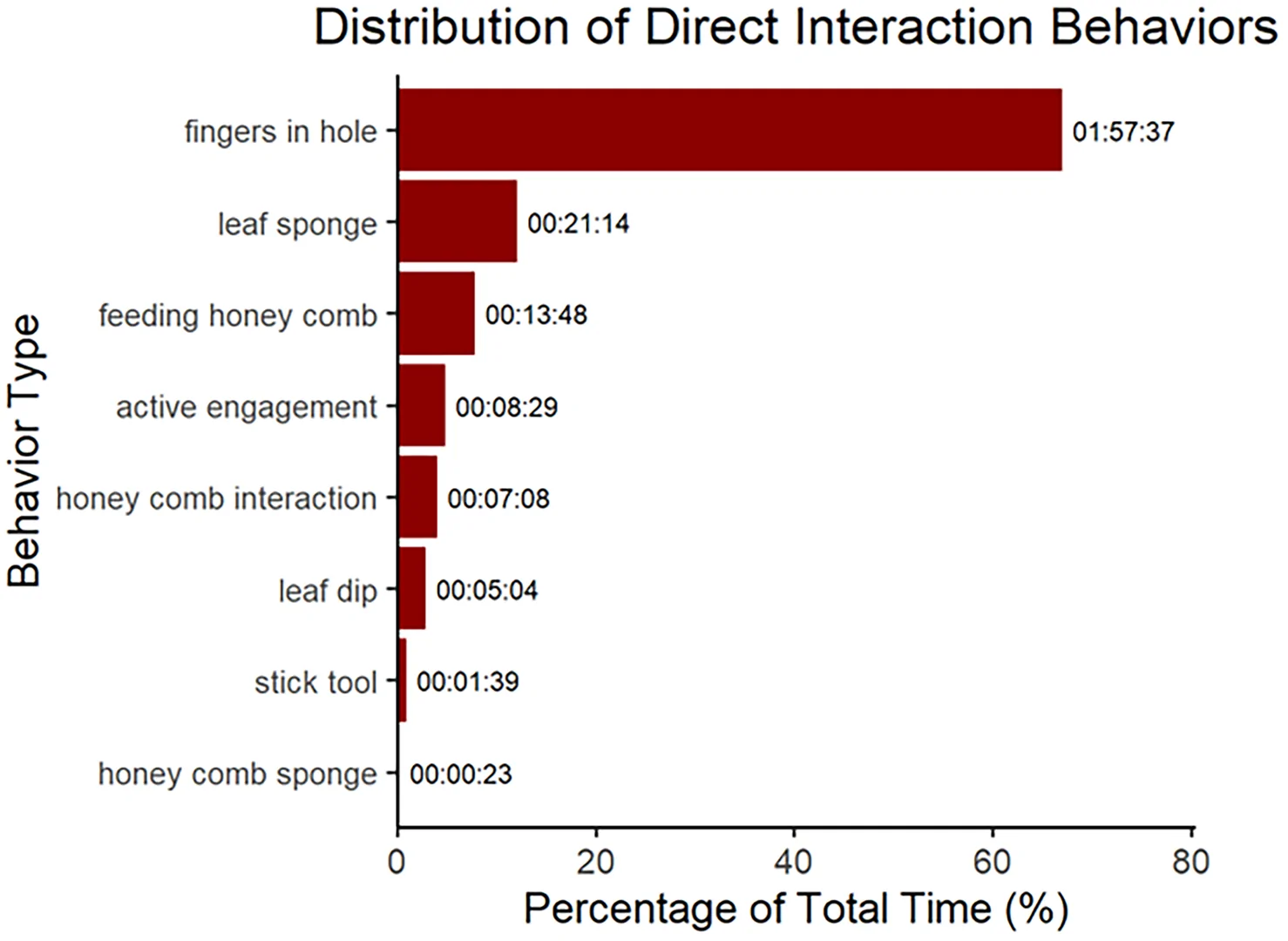
The frequency of direct interaction behaviour from the chimpanzees who engaged with the honey-trap experiment.

**Table 4.** Behaviour of the Mwera South tool users in the honey-trap experiments. For each individual, the encounter column shows the day when that individual encountered the experiment, e.g. HON started using a leaf sponge the second day when she encountered the experiment with no tool provided. All times are in hours:minutes:seconds.

experiment type	subject	encounter	total presence that day	latency to tool use	direct engagement before tool use	indirect engagement before tool use	tool type
no tool provided	ENG	1	00:29:55	00:21:40	00:18:12	00:00:53	leaf dip, stick tool
	HON	2	00:15:07	00:05:13	00:01:50	00:01:27	leaf sponge
		5	00:29:15	00:16:10	00:12:17	00:00:11	leaf sponge
		6	00:17:53	00:06:55	00:06:55	00:00:00	leaf sponge
		7	00:03:02	00:00:47	00:00:05	00:00:00	leaf sponge
		8	00:14:59	00:00:36	00:00:31	00:00:03	leaf sponge
		9	00:04:12	00:00:00	00:00:00	00:00:00	leaf sponge
	MAM	2	00:11:26	00:02:14	00:00:55	00:00:30	honeycomb sponge, leaf sponge
tool provided	ENG	1	00:23:22	00:01:14	00:01:00	00:00:01	leaf dip, leaf sponge, stick tool
		2	00:00:59	00:00:32	00:00:19	00:00:07	leaf dip
		3	01:01:44	00:03:55	00:03:42	00:00:00	leaf dip, leaf sponge, stick tool

We now describe in more detail the strategies used by the three tool-using Mwera South chimpanzees:

*Leaf tools*. As all other chimpanzees in Uganda, the Mwera South chimpanzees use a range of leaf tools customarily. In particular, the Mwera South chimpanzees regularly use leaf sponges to obtain water from holes and puddles where it collects after rain; they also use leaves as napkins to wipe themselves (see supplementary materials, videos). Experiment 1 yielded two unnamed female individuals, nicknamed HON and MAM, who manufactured and used natural sponge tools to reach the honey in the experimental log. Experiment 2 yielded one male individual, ENG, who used a sponge tool.

*Honeycomb tool*. In the same manner as a leaf sponge, one of the females, MAM, first used the honeycomb as a sponge to get the honey from the experiment log, which has never been seen before in the wild.

*Stick tools.* An adult male, ENG, manufactured a stick tool to extract honey from the experiment log. ENG used both a leafy stick to dip into the experiment hole and get honey by sucking the leaves on the end of the stick (brush tool), as well as manipulated the stick to make a bare stick with no leaves on it to dip into the hole and retrieve the honey (figure 4; supplementary material, videos). Over the course of his interactions with the experiment, ENG manufactured multiple stick tools (leafy and non-leafy) to use for honey extraction (summarized in table 4). During his engagement with experiment 2, ENG used the leafy stick that was provided to dip the leaves in and suck the honey from them. He also manufactured more stick tools and leaf sponges from nearby shrubs and saplings throughout the experiment to continue honey extraction.

**Figure 4.**
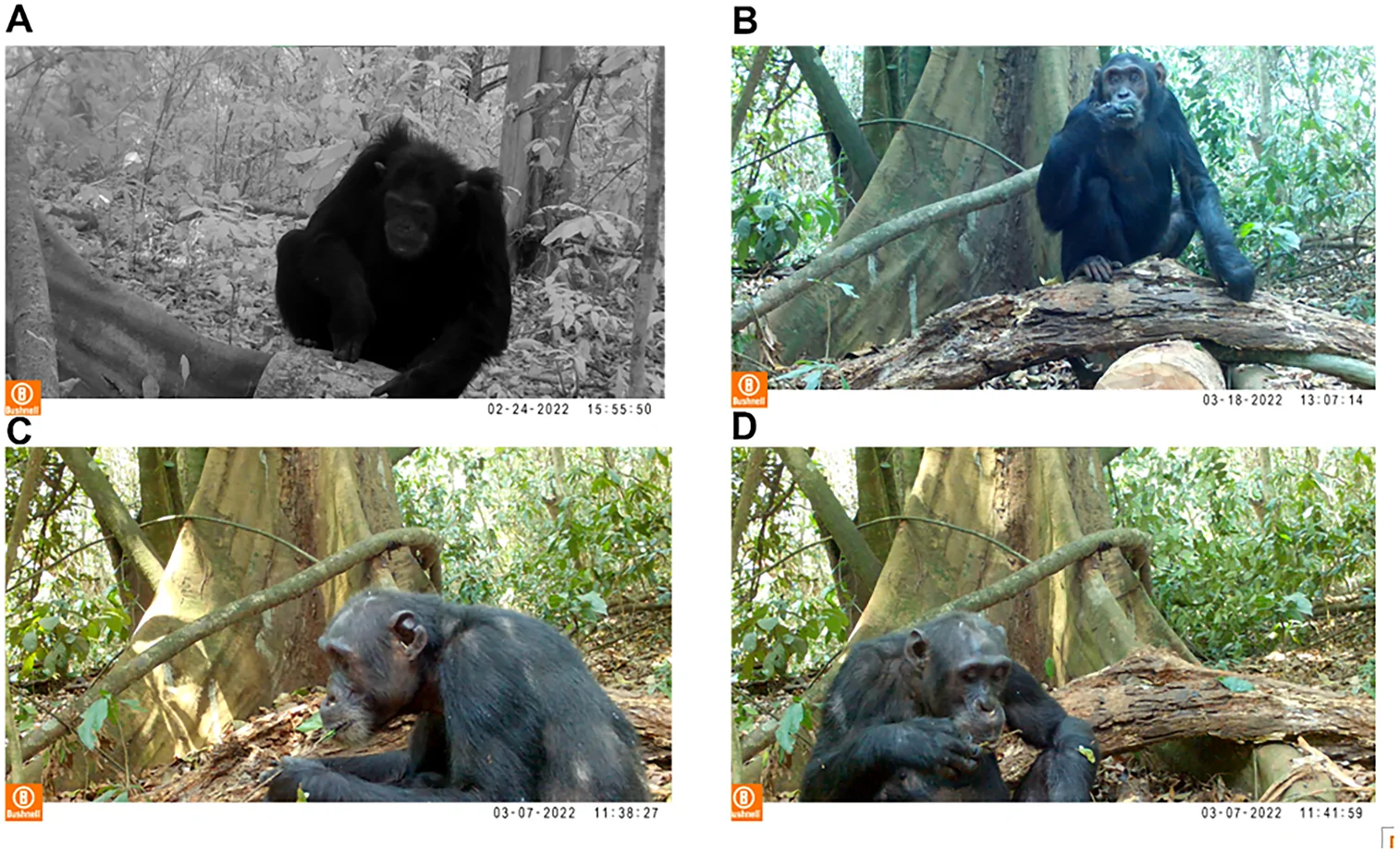
Variations in the honey-trap experiment 1 engagement. (A) An adult male using fingers to try to reach honey. (B) A female chimpanzee (HON) leaf-sponging. (C) An adult male (ENG) using the leafy stick as a dipping tool to extract honey. (D) ENG using a bare stick to extract honey. Credits: @Kelly Ray Mannion/BPCP.

## 4. Discussion

Field experiments have the potential to both uncover the tool behaviour of wild animals as well as to isolate the parts of the environment that appear salient to them. Both of these goals were pursued here, as we sought to first uncover the tool use behaviour of the Mwera South chimpanzees, and second, to determine whether individuals would recognize a potential tool for the honey extraction task. In addition, our set-up in Bugoma Forest, replicating those carried out before in Budongo and Kibale forests, allowed us to directly compare results across Ugandan communities. Overall, our comparison of chimpanzee engagement with the same field experiment across three Ugandan chimpanzee communities illuminates our understanding of cultural dynamics between and within groups, allowing us to add a conceptual difference between cultural knowledge and cultural attitude, while our results in Bugoma Forest exemplify inter-individual variation that can be found in individuals originating from the same cultural community.

### 4.1. Differences in instrumental cultural knowledge

Classical research in ethology has referred to socially acquired differences in knowledge as cultural differences [36]. In this vein, cultural differences in tool use knowledge can be evidenced by our use of the honey-trap experiment [28,37]. Indeed, this experiment has consistently found in the past that chimpanzees engaging with this experimental setting solved it with their existing instrumental cultural knowledge, i.e. leaf users used leaves and stick tool users used sticks [29]. While we may remain careful with the interpretation of our findings for the Mwera South chimpanzees because they were less habituated than the other two communities, hence possibly explaining the large observed variability in interaction, our experiments nevertheless confirm the rare sighting of stick usage prior to the experiments in Bugoma (see supplementary material and discussion below). Prior to our experimental observations, we only had admittedly weak and indirect evidence for stick tool use. Berger *et al.* [33] theorized that the Bugoma chimpanzees would be likely to use stick tools based on ecological findings that Bugoma Forest appeared more similar to Kibale Forest than to Budongo Forest. However, our results are also unique compared with the two other communities in that, remarkably and despite the comparatively small number of Mwera South individuals engaging in the honey-trap experiments, they exhibited all of the behaviour of individuals in previous studies. This includes using leaf and stick tools to solve the problem, a never-seen-before situation in over a decade of field experiments across two field sites and involving over 60 individuals. The Sonso chimpanzees indeed only ever used leaf tools to extract the honey and did not perceive the stick tool provided to them as useful, only removing the leaves from it to sponge honey. The Kanyawara chimpanzees, by contrast, who had been documented as stick tool users prior to the honey-trap experiments [38], only used stick tools to extract the honey, forgoing using the leaves attached as sponges or for dipping and using only the bare stick part when a leafy stick tool was provided. It is important to note that all Kanyawara chimpanzees routinely use leaf-sponges for water but refrained from doing so in the honey-trap experiments, leading us to conclude that the use of a tool and its contextual use can be learned separately [39]. This was seemingly the case with HON, a leaf-sponger in Mwera South who did not use any tool to extract the honey until after seeing MAM do so. A similar sequence was observed in Budongo, where PS, a male chimpanzee, engaged first with his fingers until observing RS, a female chimpanzee, leaf-sponging, and only then manufactured a leaf-sponge himself [28]. While it is beyond the scope of this article to discuss the learning mechanisms at stake, the presence of an innovator is crucial [2], with the lack of a model and subsequent observational learning potentially leading to loss of cultural behaviour in domains as different as tool use or migration routes [40,41].

### 4.2. Differences in cultural attitudes

Beyond uncovering differences in cultural knowledge, we believe our analyses comparing engagement across the three communities of Mwera South, Kanyawara and Sonso also uncover another layer of cultural differences, that is in terms of cultural attitude. In particular, the Kanyawara chimpanzees were different from the two other communities in their attitude towards honey, both in terms of engagement time as well as by having a dedicated instrumental action to extract the honey exhibited by nearly all tested individuals. Overall, Kanyawara chimpanzees might be generally more interested in exploiting the honey when finding it compared with the Mwera South and Sonso chimpanzees, although we acknowledge the limitation that this interpretation stems mainly from our 90% quantile regression model, which gives more weight to outliers. It is worth discussing here the various results highlighted by our statistical analyses, particularly as the quantile regressions at the median and at the 90% quantile zoom in on different areas of the distribution. Despite this, the two regressions both agreed in not finding any significant differences when only community was considered. This replicates the findings of Gruber *et al.* [29], but this time across three communities. Yet, the analysis in Gruber *et al.* [29] excluded two types of outliers: the chimpanzees who engaged too much with the experiment and the ones who did not engage enough (to give Sonso chimpanzees enough time for chimpanzees to use a tool, we used the median value for Kanyawara to use one (20 s) as a threshold for inclusion). Over the course of the following decade, we confirmed that giving more time—or even more opportunities to use tools—to chimpanzees in Sonso would not lead them to develop more tool usage. Rather, this relationship is inverted: chimpanzees who use tools are actually the ones who consistently spend more time with the experiment extracting honey [18]. This is replicated by our regression at the median, including more covariates, where only tool use came out as significant across three communities.

A different way to interpret our results is that an individual who engages only 2–20 s with the experiment does not actually want to engage more than this, and therefore has a limited interest in the resource, as is repeatedly the case in Sonso (*N* = 5) and Mwera South (*N* = 6). By contrast, in Kanyawara, even the low-engaging individuals engaged longer than 20 s with the experiment, and crucially, over 86% of them engaged with the same tool, sticks, to exploit this resource. On a side note, all data in Kanyawara were acquired in a matter of weeks, compared with months in Sonso and Mwera South, suggesting that the duration of engagement for the Kanyawara chimpanzees, all opportunities equal, would be much longer than for the two other communities, possibly producing more outliers too. Taking the entire spectrum of engagement with the experiments without excluding outliers is therefore equally important. This is what our full model at the 90% quantile investigated. In this model, we found a significant community effect, with the Kanyawara chimpanzees engaging longer than both the Mwera South and Sonso chimpanzees. By contrast, in this model, tool use was no longer significant, but the number of encounters, age class and community were. Across the various models, this suggests that community attitudinal differences are either influenced by tool use (cultural knowledge) or by outliers, suggesting that differences in individual motivations interfere with cultural differences in attitude.

### 4.3. Differences in individual motivation

Differences in individual motivation are best exemplified by our findings in Bugoma. While most Mwera South chimpanzees did not depart from what was observed in Sonso a decade earlier, two individuals shone by their creativity, displaying a behavioural flexibility not seen in the two other communities. First, MAM used a honeycomb to sponge the honey from the experiment. While innovation is scarce, and though seemingly difficult, it has been documented in the wild before [2,42]. We hypothesize that a chewed honeycomb resembles a fruit wedge, which is a common product of their fruit-eating diet [43]. The possibility for at least some chimpanzees to flexibly use part of their environment as tools, beyond their set function (in that case ‘food’), underlines a potential larger capacity for adaptation in wild chimpanzees beyond conservatism or functional fixedness [18]. This was particularly the case for adult male ENG. Indeed, this highly inventive male exemplifies the individual variation that can occur between chimpanzees, even from the same group, engaging in the same task. After manufacturing a stick tool on his own in experiment 1, he manufactured both stick tools and leaf-sponges from the provided leafy stick, as well as from the experimental setting, displaying an undocumented flexibility in the honey-trap experiments so far. Crucially, while trial-and-error is expected in younger individuals while they develop their tool set, this behaviour was observed in a single adult male, for whom the cultural baggage of his community should be well acquired by that time, and possibly less flexible because of functional fixedness (see [18] for discussion). While we only describe ENG's behaviour here, our model at the 90% quantile showed that adults are actually engaging more with the experiment than other age classes. A different approach here is thus to acknowledge that there are clear outliers within their own communities, who have grown particularly fond of a given resource, and who will go to a greater extent to find creative ways to obtain it because of motivation alone. Such individuals may become innovators in their own right, although it is not clear how the two characteristics interact.

### 4.4. Merging cultural attitudes, knowledge and individual motivation

Over the last decades, much attention has been put on cultural knowledge and how it is realized at the behavioural level, particularly through tool use differences [38]. The original honey-trap experiment articles are well situated within this tradition [28,29], showing in addition that community cultural knowledge will influence how they solve experimental tasks. Since then, this finding has been replicated in other species, such as capuchins [44]. In parallel to the development of this stream of research, there has been increasing evidence of cultural traditions that may extend beyond tool use traditions. Next to the historical studies of bird vocal cultural learning [45], there has been increasing discussion that diet and foraging techniques may be learned socially across taxa (birds: [46], cetaceans: [1,47]), giving ground to the idea that we have only seen the tip of the iceberg of animal cultures [10]. We believe that our study contributes to drilling into the iceberg by offering conceptual distinctions for cultural differences that may allow identifying new cultural variants in animals.

Our results suggest that the Mwera South chimpanzees do not associate stick use with honey in a cultural manner as do the Kanyawara chimpanzees, being in that respect closer to the Sonso chimpanzees in their attitude towards honey, while taking into account idiosyncratic variations of individuals of all communities connected to their motivation. As such, we believe that, in addition to documenting differences in instrumental cultural knowledge across the three communities, our results also support the existence of differences in *cultural attitudes* toward the resource, which can be considered as a separate component of culture. This proposed separation between cultural attitudes and knowledge allows refining proposed concepts in the literature, such as foraging and hunting preferences. For example, Samuni and collaborators [48] describe what they call prey preferences in bonobos, highlighting them as a potential cultural trait. We fully agree that the acquisition of such preferences can be cultural, but we believe that they do not constitute cultural knowledge *per se* (the reverse would be that every prey species for every predator would be part of their ‘cultural knowledge’, missing the social transmission requirement [49]). We also believe that the use of the wording ‘prey preference’ for a cultural trait is too broad and characterizes an individual's choice better than its community's, rather aligning with variation in individual motivation. On the contrary, ‘attitudes’ refer to a general way of being and echo, for example, the findings of Rose and collaborators [50] that three communities of white-faced capucins consistently differed in ‘*boldness, aggression and pugnacity*’*,* fuelling ‘*cross-site differences in behavior toward allospecifics*’, an attitudinal stance. While the authors stopped short of calling these differences cultural, attitudes are often cultural and semantically combined as such (‘cultural attitudes’) in the scientific literature. For our purpose, cultural attitudes then determine whether a particular prey is considered staple food in the community: it is generally consumed by the community, considered by members as part of their diet (rather than an opportunistic tasting or killing), but still allowing for variation across individuals (some individuals prefer that prey, which influences their motivation to consume it, and can lead to the instrumental learning of the best way to access it). Finally, if there is no consumption of the resource, there is either a negative or neutral attitude towards it, and probably no instrumental technique related to it, but that does not preclude individuals with new knowledge at the time to introduce interest in the community for a given substrate. For instance, Biro and collaborators [51] relate that an immigrant female knew about a certain type of nuts unknown to her new community and raised the interest of some youngsters, changing their attitude from neutral to interested in the substrate. If that attitude had stayed in the community, this could have become a new cultural attitude. Figure 5 summarizes our thinking.

**Figure 5.**
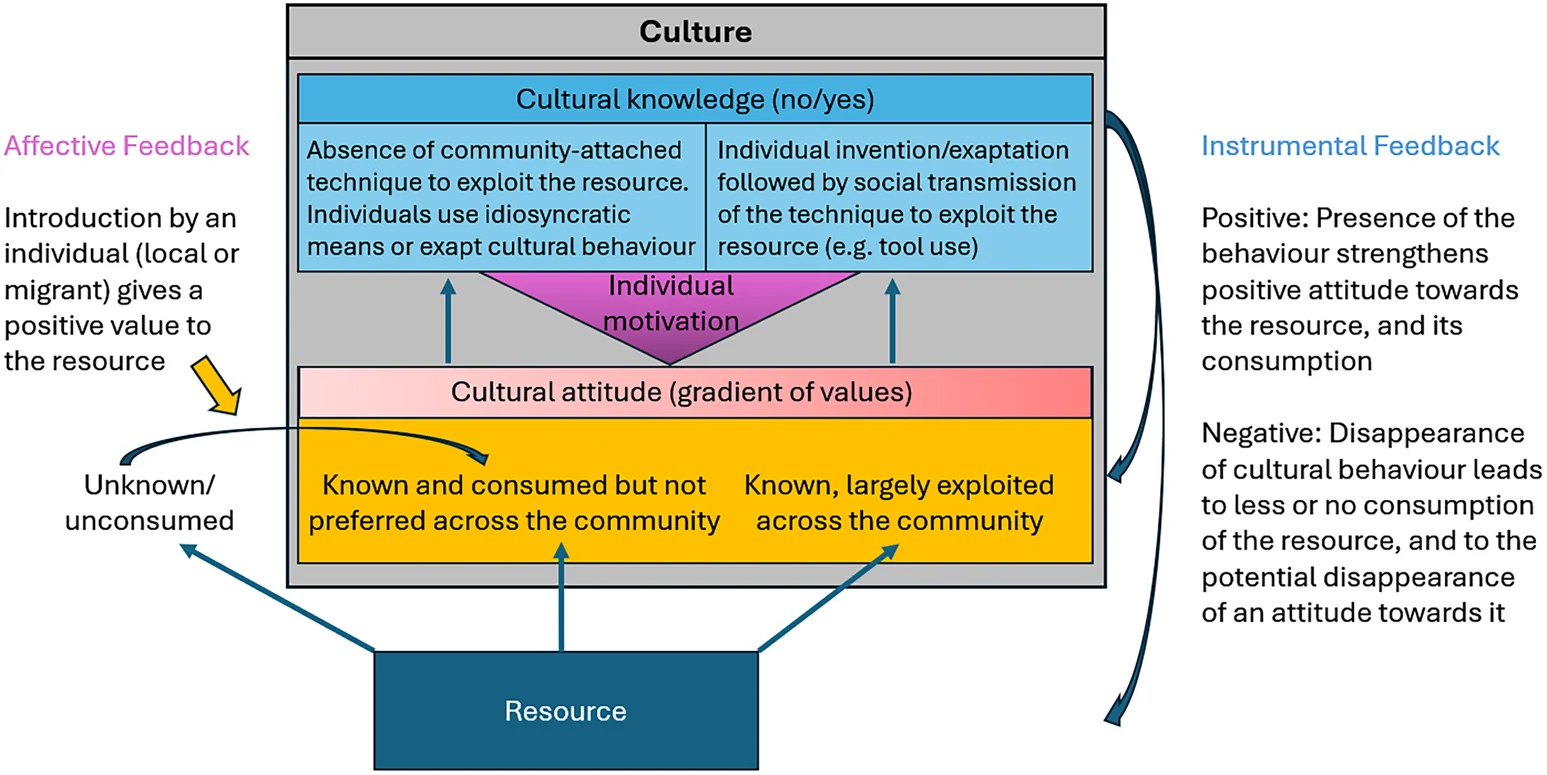
Culture as an interplay between cultural knowledge, attitude and individual motivation in the context of resource exploitation. A given resource can be variously known by a social group and can be part of its culturally transmitted diet or not (central and right arrows). In that case, the resource can be a preferred food, widely consumed across the community, leading to the innovation or exaptation of an existing cultural behaviour to exploit the resource. By contrast, if the resource is not preferred, individuals can innovate or exapt the function of an existing behaviour (cultural or not) to exploit the resource, depending on individual motivation. The exploitation of the resource can vary across time, maximal when an instrumental means is present (cultural knowledge), but minimal if this instrumental means disappears. Similarly, an individual (e.g. a migrant) can give value to an unknown resource, leading it to be included in the cultural diet of the community.

Overall, we believe the addition of our proposed conceptual layers to animal cultures is warranted to allow interpreting fine-scale attitudinal variation that may be cultural, such as found in traditions pertaining to social culture [52]. Nevertheless, we believe culture should not be split between material and social culture, as attitudinal aspects are present in both, fostered by the values displayed by others in the community [15]. Rather, we believe a useful distinction, broadly applicable in the discussion of animal cultures, is to evaluate how the two dimensions of attitude and knowledge (particularly its instrumental side) complement each other. We have argued that a community that displays a (positive) cultural attitude towards a particular resource can innovate or attach an instrumental means to access it. The accidental or intentional development of an instrumental technique to reach a resource may lead to positive feedback in the transmission of a positive attitude towards exploiting this resource. This remains to be investigated, particularly when researchers can document cultural innovation and its spread. However, conversely, the disappearance of the cultural knowledge of how to access the resource may negatively influence cultural attitude towards the exploitation of the resource. As such, we believe future research should re-evaluate whether culture in animals in a range of contexts, including foraging but not only, should be seen as attitude-based, knowledge-based or a combination of both, as we have argued here.

## Supplementary Material



## Data Availability

All analyses and raw data can be found at [53]. Supplementary material is available online [54].
